# The Audiphone and Kindred Instruments

**Published:** 1880-04

**Authors:** E. L. Holmes

**Affiliations:** Chicago


### ARTICLE IV.

THE AUDIPHONE AND KINDRED INSTRUMENTS. By E. L. HOLMES, M.D., of Chicago. (Read before the Medical Society of West Chicago.)

Any improvement in the application of a well-known principle, which tends to mitigate human suffering or ameliorate any infirmity must be a subject of interest to every physician. Such improvement if it be of advantage to but a small proportion of cases for which it seems theoretically applicable, claims careful consideration.

There have recently appeared in the daily journals of the East and the West so many notices of public demonstrations, in which it is said the deaf are made to hear by means of the audiphone, that I have thought it might be of interest to members of this society to know definitely on what basis these reports rest.

It is generally known that some patients can hear a watch, held between the teeth or in contact with some portions of the head, better than when it is placed quite near the ear.

It is not so well known that a few patients, who cannot hear loud conversation, can converse readily through the medium of a small piece of wood, twenty inches, more or less, in length, one end being held between the teeth of the patient, the other end between those of the person conversing with him. The vibrations of sound are conducted through the tissues of the head to the auditory nerve. The use of the audiphone is based on the principle involved in these experiments.

The instrument is a thin but quite large plate of black vulcanized rubber, to which is attached a handle. The whole may be said to resemble a square fan.

By means of cords and a clamp near the handle, the upper edge of the thin portion may be brought downward, causing the whole plate to curve. The fan in this form is usually placed with the concave portion turned toward the person using it, the upper edge being gently pressed against the upper (eye)teeth.

The vibrations of the fan produced by sound are conveyed through the teeth and bones to the ears.

According to my own observation and that of those upon whose testimony I can rely, the number of the deaf, who are thus practically benefited is exceedingly small. Occasionally a patient is aided in hearing to a remarkable degree.

Published statements regarding the extraordinary results in the use of the audiphone should be received with great reserve. Some of these statements are gross exaggerations. Poor patients, especially, should be cautioned not to waste their means in purchasing the instrument before they have tried it, for it will certainly, in a majority of cases, disappoint them.

I have heard of patients whose hearing was improved by the use of a sheet of bristol-board, a broad plate of wood or of a Japanese fan, bent to a proper curve (tension) and placed in contact with the teeth.

It is doubtful whether the most approved form and material for this instrument has yet been discovered.

Possibly a round fan with a delicate rim of steel, the whole so constructed as to resemble in form the natural membrana tympani (concave) might be more beneficial and graceful than that now in use.

Possibly also a piece of hard rubber or other substance attached to the disc (fan) as the malleus is attached to the membrana tympani, might be a better medium for conveying the vibrations to the teeth.

It may be that the efficacy of the audiphone (and dentaphone) may be increased in some cases by closure of the external meatus as in certain experiments with the tuning-fork.

Another instrument, the dentaphone, constructed on scientific principles, is composed of a vibrating disc enclosed in a case resembling the mouth-piece of an ordinary telephone. Attached to the center of this disc is one end of a cord; the other end of the cord is fastened to a small piece of wood. The vibration of the disc, communicated through the cord to the piece of wood held between the teeth, is conveyed to the auditory nerve.

It is stated on reliable medical authority that this contrivance compares most favorably with the audiphone.

It may not be out of place to make brief allusion to the means which have been devised to improve the sense of hearing.

These are chiefly the ordinary trumpet and the artificial membrana tympani.

The action of the trumpet is too well understood to require comment. I will only state that each patient must try for himself the various forms of the instrument and select the one with which he hears best.

The artificial membrane is usually a simple disc of rubber or fish scale, to the center of which is attached a delicate style of metal or rubber. In place of this little instrument a small pellet of cotton, properly adjusted in the external meatus is often employed. These are used not only to improve hearing but also to protect ulcerations from the air and assist in the curative process. It is remarkable to what extent these simple means will, in exceptional cases, improve hearing.

I may state that all forms of auricles, vibrators, and invisible tubes so often advertised in exaggerated terms as enabling the deaf to hear distinctly are almost absolutely valueless.

The audiphone is an invention of Mr. R. S. Rhodes, of Chicago. The dentaphone is manufactured in Cincinnati.*

---

THE PROGNOSIS OF CÆSAREAN OPERATIONS is the subject of an article by Dr. Lusk of New York in the January number of the *American Journal of Obstetrics*. In this paper Dr. Lusk, after recounting the results obtained by various well-known operators and analyzing the details given of the cases, arrives at the conclusion that the operation has been frequently performed in unhealthy maternity hospitals, and also frequently on patients that were beyond hope of recovery before the operation was commenced. He argues that it is unfair to blame the Cæsarean operation for fatal results when the conditions were so unfavorable that any operation would have gone wrong.

---

* While writing the above some weeks ago, I had read no article on the subject except the one by Dr. Turnbull, in the *Archives of Otology*. Subsequently I received a reprint of an article by Dr. C. H. Thomas, from the *Philadelphia Medical Times*.

